# Medical emergency teams are associated with reduced mortality across a major metropolitan health network after two years service: a retrospective study using government administrative data

**DOI:** 10.1186/cc11843

**Published:** 2012-10-29

**Authors:** Antony E Tobin, John D Santamaria

**Affiliations:** 1 Intensive Care Unit, St Vincent's Hospital Melbourne, PO Box 2900, Fitzroy 3065, Victoria, Australia

## Abstract

**Introduction:**

Medical emergency teams (MET) are implemented to ensure prompt clinical review of patients with deteriorating physiology with the intention of averting further deterioration, cardiac arrest and death. We sought to determine if MET implementation has led to reductions in hospital mortality across a large metropolitan health network utilising routine administrative data submitted by hospitals to the Department of Health Victoria.

**Methods:**

The Victorian admissions episodes data set (VAED) contains data on all individual hospital separations in the State of Victoria, Australia. After gaining institutional ethics approval, we extracted data on all acute admissions to metropolitan hospitals for which we had information on the presence and timing of a MET system. Using logistic regression we determined whether there was an effect of MET implementation on mortality controlling for age, gender, Charlson comorbidity diagnostic groupings, emergency admission, same day admission, ICU admission, mechanical ventilation, year, indigenous ethnicity, liaison nurse service and hospital designation.

**Results:**

5911533 individual admissions and 73,599 associated deaths from July 1999 to June 2010 were included in the analysis. 52.2% were male and median age was 57(42-72 IQR). Mortality rates for MET and non-MET periods were 3.92 (3.88-3.95 95%CI) and 4.56 (4.51-4.61 95%CI) deaths per 1000 patient days with a rate ratio after adjustment for year of 0.88 (0.86-0.89 95%CI) *P *< 0.001. In a multivariable logistic regression, mortality was associated with a MET team being active in the hospital for more than 2 years. The odds ratio for mortality in hospitals where a MET system had been in place for greater than 4 years duration was 0.90 (0.88-0.92). Mortality during the first 2 years of a MET system being in place was not statistically different from pre-MET periods.

**Conclusions:**

Utilising routinely collected administrative data we demonstrated that the presence of a hospital MET system for greater than 2 years was associated with an independent reduction in hospital mortality across a major metropolitan health network. Mortality benefits after the introduction of a MET system take time to become apparent.

## Introduction

Medical emergency teams (MET) were conceived to provide prompt clinical review of deteriorating patients with the intent of averting further deterioration, cardiac arrest and death [1]. The calling criteria chosen for MET teams have been shown to be associated with increased hospital mortality [2,3] and the intensity of activation based on these criteria is inversely associated with reduction in cardiac arrests [4,5]. In Australia and New Zealand approximately 60% of hospitals with an ICU had implemented a MET system by 2005 [6].

MET systems rely on a hospital process made up of an afferent limb, an efferent limb and a management team [7,8]. The afferent limb observes patients and identifies instability whilst the efferent limb, consisting of staff skilled in critical care, responds to and manages deterioration promptly. The management team is responsible for overseeing the process, performing audit and evaluation of all calls and providing education, feedback and quality improvement. Establishing a complex system such as this and altering staff attitudes and practice takes time, and this may in part explain some of the variation in efficacy seen in studies [9-11].

In Australia, MET teams are medically led and are distinct from critical care liaison/outreach services. The latter are nurse-led services with broad roles and varied responsibilities, which, in addition to the review of deteriorating patients, include coordination of ICU discharge, routine review of ward patients, improving communication between units, and ward education and support [12]. Such services are not subject to the same activation or administrative systems as MET, nor do they provide the immediate full services of MET but rely on a model of escalation of care [7]. In Australia, critical care liaison nurses are often members of the MET in addition to their other roles [12].

Before-and-after studies from a number of institutions support the premise that a MET reduces mortality [13-15], although the only randomised multicentre trial looking at the effect of MET on mortality failed to show a benefit [16], and two recent meta-analyses questioned their effect on hospital mortality [17,18]. Using routinely collected hospital administrative data we have previously reported that the introduction of a MET at our institution was associated with a reduction in all-cause hospital mortality over a number of years [19]. The study demonstrated that it took two years for the MET to have a statistically significant effect on hospital mortality. These findings from administrative data were supported by similar results from analysis of prospective data collected according to the MERIT protocol [16] over the same time period. As suggested by others [10], it takes time to change the culture and processes in a hospital and as a result changes in mortality may take years to become apparent. Studies of MET intensity have demonstrated that MET calls increase over time and that there is an association between benefit and the number of calls, again suggesting that the greater the adoption of a MET ethos over time (reflected by call rate), the greater the institutional benefit [5,19].

Large medical administrative databases are increasingly being used to examine health care quality and the association between interventions and patient outcomes [20,21]. In Victoria Australia, administrative data are submitted by all hospitals for government audit and funding purposes. This database is known as the Victorian Admissions Episodes Data Set (VAED) [22] and its utility for monitoring care has been demonstrated [23,24]. As we had used these data in our recent study [19], we sought to determine if the implementation of METs across a large metropolitan health network (Melbourne, Victoria) would lead to a significant reduction in mortality and whether changes in mortality took time to occur.

## Materials and methods

The study was undertaken at St Vincent's Hospital Melbourne, Victoria, Australia, and ethics approval for the study was obtained from the Hospital Research and Ethics committee of St Vincent's Hospital Melbourne.

Each year the Department of Health Victoria distributes a de-identified subset of the VAED to each Victorian Hospital. The data contain demographic details, reasons for and type of admission to hospital as well as up to 40 diagnostic (ICD-10-AM) and 40 procedure codes. We used these files to extract data for a 10-year period on all acute admissions to public hospitals, for which we had information on the presence and timing of a MET system in a metropolitan area, including the state capital Melbourne and a large satellite city, Geelong. The combined population of these cities was approximately 4,255,000 in June 2010 and their combined health services also provide specialised services for the state of Victoria, which has a total population of about 5.5 million. Data on the timing and presence of a MET team and ICU liaison nurse outreach service was determined by contacting the Directors of Intensive care at the relevant hospitals. Information on the nature of the teams, their calling criteria and oversight and audit processes was not determined. A categorical variable for MET was defined as the absence of MET and the presence of MET for < 2yrs, 2 to 4 years or > 4 years. The presence or absence of an ICU liaison service was included as a binary variable.

The data in the VAED is de-identified. Variables for age, gender, ICD-10-AM codes, admission hospital, length of stay, ICU admission, mechanical ventilation, financial year of admission and indigenous ethnicity were extracted. These diagnostic codes were used to determine the comorbidity elements of the Charlson comorbidity index [25] as a means of adjusting for disease severity. To account for secular changes in care over the time period of the study, a variable for year was included in all multivariable models.

Continuous data were summarised as mean ± SD if approximately normally distributed or median and interquartile range (IQR) if skewed. Estimates are shown with 95% confidence intervals (CI). Categorical variables were reported as counts and proportions with differences assessed by the chi-square and Fisher's exact test as appropriate. Mortality rates per 1,000 inpatient days were calculated and Mantel-Haenszel methods used to determine rate ratios. Logistic regression was used to determine whether there was an independent association of MET implementation with mortality. *P*-values < 0.05 were considered significant. Statistical analysis was performed using Stata 12 (StataCorp, College Station, TX, USA).

## Results

**Figure 1:**
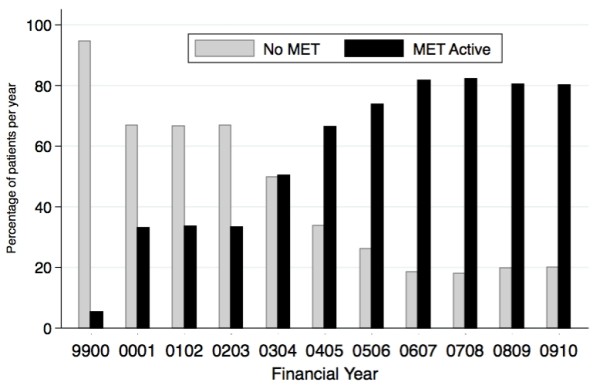
**Percentage of patients in hospitals with an active medical emergency team (MET) by financial year**.

From July 2000 to June 2010 there were 5,911,533 individual admissions and 73,599 (1.25%) deaths; 3,375,923 (57.1%) were coded as same-day admissions. Among the total admissions, 52.2% of patients were male and the median age was 57 (IQR 42 to 72) years. The patients came from twelve hospitals, six of which were designated tertiary and six metropolitan. The median number of admissions per hospital per year was 42,788 (IQR 34,158 to 52,694). One of the hospitals had an active MET at the beginning of the study period and by the end of the study period all but three hospitals had an active MET. The percentage of patients exposed to a MET increased from 5.3% at the commencement of the study to approximately 80% of patients in the final 4 years (Figure 1).

The overall mortality rate was 4.18 (95% CI 4.15, 4.21) per 1,000 patient days. Unadjusted mortality decreased in association with the introduction of a MET (Table 1). The univariate odds for death were lower in the first 2 years of MET compared with baseline and dropped further in subsequent years. Mortality rates (95% CI) for MET and non-MET periods were 3.92 (3.88, 3.95) and 4.56 (4.51, 4.61) deaths per 1,000 patient days with a rate ratio after adjustment for year of 0.88 (0.86, 0.89) *P *< 0.001 (Table 1).

**Table 1:** Unadjusted mortality and odds ratios (OR) for death by presence and duration of a medical emergency team (MET)

Patient deaths and odds ratios for death by MET implementation year				
**MET Duration**	**Alive number (%)**	**Died number (%)**	**Total, number of patients**	**OR for death (95% CI)**

Nil	2,363,881 (98.6)	33,425 (1.4)	2,397,306	Base
0 to 2 yrs	781,399 (98.8)	9,271 (1.2)	680,836	0.84 (0.82, 0.86)
3 to 4 yrs	898,356 (98.9)	10,250 (1.1)	908,606	0.81 (0.79, 0.83)
> 4 yrs	1,794,298(98.9)	20,653 (1.1)	1,814,951	0.81 (0.80, 0.83)
Total	5,837,934 (98.8)	73,599 (1.3)	5,911,533	

Univariable analysis demonstrated an association between mortality and the presence of a MET, the presence of a liaison service, year, age, ICU hours, mechanical ventilation, emergency admission, hospital level and Charlson diagnostic groups (results not shown). These variables were included in a multivariable logistic regression model. In the multivariable model, mortality was associated with patient age, year of admission, gender, ICU admission, mechanical ventilation, hospital designation, emergency admission, multiday admission, Charlson comorbidity indices and the presence of a MET team for 2 or more years (Table 2). Mortality in the first 2 years in which a MET system was in place was not statistically different from the pre-MET period, with an odds ratio (OR) of 0.99 (95% CI 0.97, 1.02). The OR for mortality where a MET system had been in place for 2 to 4 years duration was 0.93 (0.91, 0.96), and was 0.90 (0.88, 0.92) where a MET system had been in place for more than 4 years. Mortality was not associated with the presence of an ICU liaison service in the multivariable model (OR 1.00, 95% CI 0.98, 1.02). The area under the receiver-operator characteristic curve for the model was 0.94. The Hosmer-Lemeshow statistic was significant (*P *< 0.001).

**Table 2:** Results of multivariable logistic regression

		Odds ratio	*P*-value	95% Confidenceintervals	
	
				Lower	Upper
MET (pre-MET base)	MET 0 to 2 yrs	0.99	0.553	0.96	1.02
	MET 2 to 4 yrs	0.93	< 0.001	0.91	0.96
	MET > 4 yrs	0.90	< 0.001	0.88	0.92
Year (1999 to 2000 base)	2000-01	1.16	< 0.001	1.11	1.20
	2001-02	1.67	< 0.001	1.61	1.74
	2002-03	1.10	< 0.001	1.06	1.14
	2003-04	1.06	< 0.01	1.02	1.10
	2004-05	0.95	< 0.05	0.92	0.99
	2005-06	0.91	< 0.001	0.87	0.94
	2006-07	0.87	< 0.001	0.84	0.91
	2007-08	0.90	< 0.001	0.86	0.93
	2008-09	0.89	< 0.001	0.86	0.93
	2009-10	0.81	< 0.001	0.77	0.84
Age (< 40 yrs base)	40 to 49	1.67	< 0.001	1.58	1.77
	50 to 59	2.31	< 0.001	2.19	2.42
	60 to 69	3.33	< 0.001	3.18	3.48
	70 to 79	5.52	< 0.001	5.29	5.77
	80+	11.99	< 0.001	11.49	12.52
Gender	Male	1.05	< 0.001	1.03	1.06
Hospital level (base = metropolitan)	Tertiary	0.87	< 0.001	0.85	0.88
Multiday admission	Yes	1.84	< 0.001	1.80	1.89
ICU admission	Yes	3.40	< 0.001	3.27	3.53
Ventilation	Yes	4.57	< 0.001	4.38	4.76
Emergency admission	Yes	9.18	< 0.001	8.90	9.46
Acute myocardial infarction	Yes	2.14	< 0.001	2.08	2.19
Congestive cardiac failure	Yes	2.36	< 0.001	2.31	2.41
Peripheral vascular disease	Yes	2.18	< 0.001	2.10	2.25
Cerebrovascular disease	Yes	4.18	< 0.001	4.06	4.30
Dementia	Yes	2.08	< 0.001	2.02	2.15
Chronic obstructive airways disease	Yes	1.51	< 0.001	1.47	1.55
Rheumatoid arthritis	Yes	1.46	< 0.001	1.31	1.61
Peptic ulcer disease	Yes	1.09	< 0.05	1.02	1.17
Mild liver disease	Yes	1.39	< 0.001	1.31	1.48
Hemi/paraplegia	Yes	1.28	< 0.001	1.23	1.33
Chronic kidney disease	Yes	1.70	< 0.001	1.67	1.74
Cancer	Yes	3.31	< 0.001	3.22	3.41
Mod/severe liver disease	Yes	7.09	< 0.001	6.68	7.53
Metastatic cancer	Yes	2.89	< 0.001	2.79	2.99
AIDS	Yes	4.85	< 0.001	4.11	5.72
Indigenous ethnicity	Yes	1.44	< 0.001	1.28	1.61

MET, medical emergency team.

To estimate the potential effect of a mature MET we used the mortality rate for the pre-MET cohort of 1.39% and the odds ratio of 0.90 for a MET system of > 4 yrs duration, to calculate an estimated mortality rate under a mature MET system. The model mortality so calculated was 1.25% or an absolute reduction of 0.14%. This translates to approximately one life saved for every 714 patients exposed to a hospital with a mature MET team, or approximately 56 lives saved per year in a hospital with 40,000 admissions per year.

## Discussion

Using routinely collected administrative data we have demonstrated an independent association between the implementation of a hospital MET and a reduction in mortality across a large metropolitan health system. The mortality benefit was delayed, only becoming apparent after the MET had been active for longer than 2 years. This is consistent with the MET system taking time to change the culture and processes of a hospital as we have demonstrated previously [19].

The process of MET is complex, incorporating three interdependent limbs [7,8]. The afferent limb reporting instability consists largely of nursing staff and junior doctors. For this limb to work requires education and acceptance by the staff. Cretikos *et al. *[11] interviewed staff from the MERIT study hospitals and found that there was substantial variation in MET use between hospitals, and this related not only to understanding the principles of MET systems, but also to positive perceptions of the MET. Nurses' engagement with the MET has been shown to be influenced by their level of training in it, their clinical experience and support both from the MET and the ward teams [26]. Buist *et al. *[27] reported that the efficacy of the team and service can be improved by education and training. Such processes will take time to implement and refine, perhaps relying on positive reinforcement and education by the attending team and feedback from the hospital management [28].

The dose of MET is felt to be important for effect. Jones *et al. *[5] reported an inverse relationship between call intensity and the reduction in cardiac arrests, as have others [19]. As call rates tend to increase over time [5,19], it is understandable that there may be a delay between implementation of a MET and mortality benefits. Even then, barriers may persist that limit MET activation relating to other system problems such as competence, knowledge of disease, hierarchy, handovers and circumstance [29,30], and interventions may be needed to improve call rates [31]. Therefore it is not surprising that the MERIT study [16] and some other short-term comparison studies failed to show a mortality benefit of the MET. The findings from this study on the time course of effect agree with findings from our institution, which demonstrated that it took time for mortality to reduce significantly after a MET was introduced [19].

How the team is composed is likely to affect the efferent limb. Traditionally, both ICU medical and nursing staff have led teams in Australia. However, alternative structures have been used elsewhere, including nurse- and respiratory technician-led teams [32]. We do know that in our study all the teams were medically led and this is important given the paucity of evidence supporting nurse-led teams [5,32,33]. Although we know that all teams by definition included medical staff, we lack information on the exact nature of teams, their calling criteria and their oversight so are unable to draw conclusions about the importance of how the team is managed [34]. Variation in team makeup and management would likely lessen any effect of the MET and as such our estimate of effect is perhaps a more pragmatic one.

This study focused on mortality as a measure of the effect of the MET but this may underestimate the true impact. MET systems were initially introduced to reduce cardiac arrests. This may occur by intervening early to prevent arrest or by identifying those patients with failing physiology who will not benefit from advanced life support and implementing not for resuscitation (NFR) orders. Both may result in a reduction in cardiac arrest calls but only the former will lead to a reduction in hospital mortality. Furthermore, MET may have other benefits such as preventing deterioration and complications, preventing ICU admissions, ward education and staff satisfaction [30], and 'changing the journey not the outcome' [35], such as when NFR orders are put in place. Therefore, mortality data may only reflect a small part of the total beneficial effects of MET.

This study is limited by its retrospective nature, dependence on reliable coding across many years and institutions, and the limited data with which to adjust for individual patient differences. Trained data extractors collect VAED data and the information is used to code all hospital separations. It is submitted to the Department of Health Victoria and forms the basis of funding for Victorian public hospitals. Furthermore, it is internally and externally audited at regular intervals to assess completeness and accuracy. Its use for the study can be criticised for not being purpose-collected, however, it does contain parameters that are known to be associated with outcome and we have previously shown that use of these data yields similar results to analysis of prospective purpose-collected data [19].

Considerable changes in health systems will have occurred concurrently with the introduction of MET, including critical care liaison/outreach. The duties of critical care outreach teams overlap with that of MET and may be additive to any outcome benefit associated with MET. Studies of ICU outreach have shown variable results but they have been associated with reductions in adverse events, ICU readmission and mortality, as well as improved nursing confidence and knowledge [36]. Given this, our model controlled both for the presence of a critical care liaison service and year to limit confounding due to secular changes in health care over the study period. We found no evidence for an association between the presence of a critical care liaison service and mortality, although there were significant reductions in mortality in association with year.

The Hosmer-Lemeshow goodness of fit-statistic for the logistic model suggested poor calibration. However, Kramer and Zimmerman [37] studied the effect of sample size on this statistic and found that for studies with large numbers the statistic was often significant, and that other measures be used for validation. The dataset used for this study has 5,911,533 individuals with an incidence of the event of interest, mortality, of only 1.25%. Looking at individual groups of predicted probability the model was satisfactory where the predicted probability of death was > 0.005. Below this the model overestimated deaths substantially.

## Conclusions

Using routinely collected administrative data we have shown that the implementation of MET systems at individual hospitals was associated with a reduction in hospital mortality across a large metropolitan health system. This effect took time to become apparent, consistent with the assumption that the MET system takes time to alter hospital culture and process. Given that randomised trials of MET are unlikely to occur in the future, administrative data may provide a useful tool for building the evidence base for MET both at institutional and health network levels.

## Key Messages

• Mortality across a large metropolitan health service decreased in association with the introduction of medical emergency teams

• Change in mortality took time to become apparent

• Delayed reduction in mortality is consistent with MET implementation taking time to change hospital process and culture

• Routinely collected government administrative data may be useful to monitor changes to health service process

## Abbreviations

CI: confidence interval; IQR: interquartile range; MET: medical emergency team(s); NFR: not for resuscitation; OR: odds ratio; VAED: Victorian admissions episodes dataset.

## Competing interests

The authors declare that they have no competing financial or non-financial interests.

## Authors' contributions

AT performed the statistical analysis and drafted the manuscript. JS conceived the study concept, extracted and cleaned the data and helped draft the manuscript. Both authors have read and approved the manuscript for publication.
